# Deciphering the Nexus: Exploring Learning Styles and Academic Success Among Medical Students Through a Comprehensive Study

**DOI:** 10.7759/cureus.59079

**Published:** 2024-04-26

**Authors:** Rajeswari Kathiah, Praveena Daya A, Sathish Selvakumar, Kunnumbrath Arathi, Meenakshisundaram K

**Affiliations:** 1 Pathology, All India Institute of Medical Sciences, Madurai, Madurai, IND; 2 Community Medicine, Tirunelveli Medical College, Tirunelveli, IND; 3 Pathology, Employees' State Insurance Corporation Medical College and Post Graduate Institute of Medical Sciences and Research, KK Nagar, Chennai, IND

**Keywords:** pedagogical approaches, student achievement, curriculum design, learning preferences, higher education, teaching methodologies, educational research, medical students, academic success, learning styles

## Abstract

In the dynamic landscape of medical education, recognizing and catering to the diverse learning styles of students are pivotal for fostering academic success. This study investigates the intricate relationship between learning styles and academic performance among medical students. A sample comprising 201 second-year Bachelor of Medicine and Bachelor of Surgery (MBBS) students from two batches participated in this cross-sectional study. Utilizing the Grasha-Riechmann Student Learning Style Scales, students were categorized into six distinct learning styles: independent, avoidant, collaborative, dependent, competitive, and participatory. Academic performance was assessed through cumulative scores at the end of the academic year. Statistical analyses, including descriptive statistics, Spearman's correlation analysis, and the Kruskal-Wallis H test, were conducted using IBM SPSS Statistics for Windows, Version 25, (Released 2017; IBM Corp., Armonk, New York, United States).

The findings revealed a rich diversity of learning styles among medical students, with independent learning emerging as the most prevalent style. However, intriguingly, no statistically significant difference in academic performance was discerned across the various learning styles. Nonetheless, correlation analysis uncovered weak positive correlations between independent, dependent, and participatory learning styles with academic performance, while an equally weak negative correlation was observed for the avoidant style. These results underscore the necessity for tailored educational strategies that can accommodate the heterogeneous learning preferences exhibited by medical students. While certain learning styles may be favoured by students, their adoption does not guarantee academic success. Thus, educators are urged to embrace flexible teaching methodologies to accommodate the diverse learning styles present within medical education, ultimately fostering student engagement and achievement. This study illuminates the imperative of understanding and addressing diverse learning styles among medical students, laying the foundation for further research into optimizing teaching methodologies in medical education.

## Introduction

In the multifaceted realm of medical education, the importance of acknowledging and accommodating diverse learning styles among students cannot be overstated. The intricate interplay between how students prefer to learn and their academic performance is a topic of paramount significance for educators and institutions alike [1]. This study endeavours to delve into this critical relationship, shedding light on how learning styles influence the academic journey of medical students.

Medical education, renowned for its rigorous and demanding curriculum, presents a unique set of challenges for both students and educators. Amidst the vast expanse of medical knowledge and the ever-evolving landscape of healthcare, the ability to grasp and assimilate information effectively is indispensable for success. Central to this process is the recognition that students exhibit a myriad of learning styles, each with its strengths, preferences, and nuances [2].

There are six different types of learners based on their learning styles: independent, avoidant, collaborative, dependent, competitive, and participatory. Understanding these learning styles is fundamental for educators seeking to optimize the teaching and learning experience. By tailoring instructional methods to align with the diverse preferences of students, educators can foster an environment that nurtures engagement, retention, and ultimately, academic achievement [3]. However, the extent to which learning styles impact academic performance within the context of medical education remains an area ripe for exploration [4,5].

This study aims to bridge this gap by investigating the relationship between learning styles and academic performance among medical students. Through the utilization of robust assessment tools such as the Grasha-Riechmann Student Learning Style Scales (GRLSS), we seek to delineate the spectrum of learning styles prevalent among medical students [6,7]. Furthermore, by examining academic performance metrics, such as cumulative scores at the end of the academic year, we endeavour to elucidate the extent to which learning styles influence academic outcomes.

By unravelling the intricate dynamics between learning styles and academic performance, this study seeks to inform educational practices and pedagogical approaches within medical education. Through a nuanced understanding of how students learn best, educators can tailor their strategies to accommodate diverse learning preferences, thereby fostering an inclusive and supportive learning environment.

Objectives

The first objective was to identify the predominant learning styles among second-year Bachelor of Medicine and Bachelor of Surgery (MBBS) students utilizing the GRLSS. The second objective was to explore the potential correlation between the identified learning styles and the academic performance of the students.

## Materials and methods

Methodology

Study Design and Setting

This study adopted a cross-sectional design to investigate the relationship between learning styles and academic performance among medical students. Cross-sectional studies are well-suited for examining associations between variables at a single point in time, making them ideal for exploring the intricate dynamics between learning styles and academic outcomes. The study was conducted at the Department of Pathology, Employees' State Insurance Corporation (ESIC) Medical College, Chennai, India.

Pilot Study

Prior to the commencement of the main study, a pilot study was conducted involving 30 volunteer participants from the third phase of the medical curriculum. The purpose of the pilot study was to evaluate the feasibility and refine the methodology of data collection procedures. Participants were selected using convenience sampling methodology from the same academic setting, ESIC Medical College, Chennai.

Data collected from the pilot study were subjected to preliminary analysis to identify any potential issues or challenges in the study procedures. Feedback from participants regarding the clarity of instructions, the time required for completion, and overall experience with the assessment tools was gathered and incorporated into the final study design.

Participants

The study sample comprised 201 second-year MBBS students from two batches, ensuring a diverse representation of the student population.

Sample Size Calculation

A minimum sample size of 156 students was determined to provide adequate statistical power for detecting significant associations between learning styles and academic performance. This was calculated based on a power analysis using a significance level (α) of 0.05, desired power (1 - β) of 0.80, and an estimated effect size derived from prior research or pilot studies. Additionally, practical constraints and the feasibility of data collection were considered in determining the sample size.

Inclusion Criteria

It was mandatory for prospective participants to take part in the study voluntarily after being adequately informed about its purpose, procedures, potential risks, and benefits.

Exclusion Criteria

Individuals who were unable or unwilling to provide written consent to participate in the study were excluded. Students who failed to complete two consecutive assessments, as required for the study protocol, were excluded from the analysis to ensure data consistency and reliability. Inclusion criteria stipulated that participants must provide written informed consent, while exclusion criteria precluded students who were unable to complete two consecutive assessments.

Sampling

Convenience sampling methodology was employed to recruit participants, leveraging the accessibility and availability of students within the medical college setting.

Study Tool

The GRLSS served as the primary tool for assessing learning styles among participants. These scales categorize students into six distinct learning styles: independent, avoidant, collaborative, dependent, competitive, and participatory. The utilization of validated assessment tools ensures the reliability and validity of the data collected (18).

Data Collection Procedure

Data collection involved administering the GRLSS in the printed format to participants to assess their predominant learning styles, at the beginning of phase two of the medical curriculum. Prior to the administration, the students were given orientation towards the scale. Additionally, academic performance data, represented by cumulative academic scores obtained through theory and practical examinations conducted throughout the year in the Department of Pathology were gathered from departmental records. On average, there were six theory-based assessments and three practical-based assessments for each batch; the scores of these tests varied, and the final score in % was recorded. To uphold the integrity of the study findings and minimize potential biases, uniformity in teaching methodologies and assessment protocols was maintained across both batches of second-year MBBS students. This involved consistent delivery of lectures, seminars, and practical sessions, as well as standardized procedures for theoretical and practical examinations within the Department of Pathology. This approach aimed to ensure that any observed differences in academic performance could be attributed to learning styles rather than variations in teaching or evaluation methods. 

Statistical Analysis

Statistical analysis was conducted using IBM SPSS Statistics for Windows, Version 25, (Released 2017; IBM Corp., Armonk, New York, United States) to analyze the collected data. Descriptive statistics, including frequency distributions and percentages, were employed to summarize demographic characteristics and learning style preferences among participants. Correlation analyses, such as Spearman's correlation analysis, were utilized to examine the relationship between learning styles and academic performance. Furthermore, the Kruskal-Wallis H Test was employed to assess differences in academic performance scores across different learning styles.

Ethical Considerations

Ethical approval was obtained from the institutional review board (IRB) to ensure the protection of participants' rights and confidentiality. Informed consent was obtained from all participants before their inclusion in the study, and measures were implemented to safeguard their privacy and anonymity throughout the research process.

## Results

The results of the study provided a comprehensive overview of the learning styles prevalent among second-year MBBS students and their correlation with academic performance. Through meticulous analysis of participant demographics, preferred learning styles, and academic achievements, key patterns and associations emerged, shedding light on the nuanced dynamics of learning within this cohort.

Batch of Study Participants

The study comprised 201 second-year MBBS students, with 106 (52.7%) from the 2021 batch and 95 (47.3%) from the 2022 batch (Table 1).

**Table 1:** Batch of study participants (n=201)

Batch	Number of participants	Percentage
2021 batch	106	52.7
2022 batch	95	47.3
Total	201	100.0

Gender Distribution

Among the participants, 98 (48.8%) were males and 103 (51.2%) were females (Table 2).

**Table 2:** Gender distribution among study participants (n=201)

Gender	Frequency	Percent
Males	98	48.8
Females	103	51.2
Total	201	100.0

Preferred Learning Styles

Among the diverse array of preferred learning styles observed among the second-year MBBS students, independent learning emerged as the predominant choice, with nearly half of the participants endorsing this approach. Participant learning style followed closely, suggesting a significant inclination towards interactive and engaged learning methods. Collaborative and dependent styles also garnered notable representation, highlighting the varied learning preferences within the cohort. Among the 201 study participants, 94 (46.8%) were independent learners, 36 (17.9%) were participant learners, 25 (12.4%) were collaborative learners, 21 (10.4%) were dependent learners and 13 (6.5%) and 12 (6%) were competitive and avoidant learners, respectively (Table 3).

**Table 3:** Preferred Learning styles among study participants (n=201)

Learning style	Frequency	Percent
Avoidant	12	6.0
Collaborative	25	12.4
Competitive	13	6.5
Dependent	21	10.4
Independent	94	46.8
Participant	36	17.9
Total	201	100.0

Cross-Tabulation of Learning Styles and Batches

Cross-tabulation between the year of study and learning styles showed that there was a statistically significant difference between the batches and learning styles (p-value- 0.02). In the 2021 batch of students, 41 (38.7%) preferred the independent learning style followed by 24 (22.6%) preferring the participant style. The majority of the 2022 batch of students, 53 (55.8%), preferred an independent learning style followed by 15 (15.8%) preferring a collaborative learning style (Table 4).

**Table 4:** Cross-tabulation between the two batches of students and preferred learning styles (n=201)

Batch	Avoidant		Collaborative		Competitive		Dependent		Independent		Participant		Total		X2 value	p-value
	N	%	N	%	N	%	N	%	N	%	N	%	N	%	12.9	.02
2021	9	8.5	10	9.4	7	6.6	15	14.2	41	38.7	24	22.6	106	100		
2022	3	3.2	15	15.8	6	6.3	6	6.3	53	55.8	12	12.6	95	100		

GRLSS Sub-Scale Scores

The GRLSS subscale scores revealed distinct preferences among the second-year MBBS students. Collaborative learning style emerged with the highest mean score, (mean ± standard deviation (SD) = 2.98 ± 1.25) indicating a strong inclination towards teamwork and cooperative learning methods. In contrast, the competitive learning style exhibited the lowest score, (mean ± SD = 2.51 ± 0.78) suggesting a lesser preference for competitive academic environments among the participants (Table 5).

**Table 5:** GRLSS sub-scale scores of the students (n=201) GRLSS: Grasha-Riechmann Student Learning Style Scales

Learning style	Scores		
	Mean ±SD	Minimum	Maximum
Independent	2.98 ±1.25	1	5.0
Avoidant	2.71±.71	.5	4.6
Collaborative	3.87±.73	.5	5
Dependent	3.53±.63	.4	4.6
Competitive	2.51±.78	.3	4.9
Participatory	3.46±.65	.5	5.0

Academic Performance

The mean scores of the students following participant learning were high (Theory exam scores: mean ± SD = 45.22 ± 13.40. After the participant learning style, students in the independent learning style scored high marks (mean ± SD = 44.46 ± 14.83) (Table 6).

**Table 6:** Academic performance of students following various learning styles

Learning style		Marks (out of 100)
Avoidant (n=12)	Mean	40.83
	Standard deviation	12.503
	Minimum	16
	Maximum	59
Collaborative (n=25)	Mean	43.48
	Standard deviation	12.965
	Minimum	14
	Maximum	66
Competitive (n=13)	Mean	41.69
	Standard deviation	13.092
	Minimum	14
	Maximum	65
Dependent (n=21)	Mean	41.95
	Standard deviation	14.55
	Minimum	25
	Maximum	70
Independent (n=94)	Mean	44.46
	Standard deviation	14.83
	Minimum	6
	Maximum	81
Participant (n=36)	Mean	45.22
	Standard deviation	13.41
	Minimum	21
	Maximum	83

Correlation Between Learning Style Scores and Academic Performance

A statistically significant weak positive correlation was observed between academic performance and the following learning styles scores, independent score (p = 0.004, r = 0.200), dependent score (p=.002, r=0.216), and participatory score (p < 0.001, r = .0251). A weak negative correlation was found between academic performance scores and avoidant scores (p = 0.019, r = − 0.165) (Table 7).

**Table 7:** Correlation between learning style scores and academic performance *r values and p-values are based on Spearman's correlation analysis

Academic performance	Independent	Avoidant	Collaborative	Dependent	Competitive	Participatory
r value*	.200	-.165	.103	.216	.107	.251
p-value*	0.004	.019	.145	.002	.130	<0.001
n	201	201	201	201	201	201

Comparison of Academic Performances Among the Learning Style Groups

Weak positive correlations were observed between academic performance and independent, dependent, and participant learning style scores. A weak negative correlation was found between academic performance and avoidant learning style scores. A Kruskal-Wallis H Test showed that there was no statistically significant difference in academic performance scores between the students who follow different learning styles, X2(5) = 4.23, p = 0.517 (Table 8).

**Table 8:** Comparison of academic performances among the learning style groups (n=201)

Academic performance	Independent median (Min-Max)	Avoidant median (Min-Max)	Collaborative median (Min-Max)	Dependent median (Min-Max)	Competitive median (Min-Max)	Participatory median (Min-Max)	Kruskal-Wallis p-value
	44 (6-81)	41 (13-59)	43 (14-66)	36 (25-70)	42 (14-65)	45 (21-83)	.517

## Discussion

The findings of this study provide valuable insights into the relationship between learning styles and academic performance among medical students. The discussion below synthesizes the results obtained and contextualizes them within the broader framework of medical education.

Diverse Learning Styles

The results revealed a rich diversity of learning styles among medical students, with independent learning emerging as the most prevalent style. This observation underscores the heterogeneous nature of the student population and highlights the importance of recognizing and accommodating varied learning preferences within medical education. The prevalence of independent learning may reflect a shift towards self-directed learning among medical students, a trend that aligns with contemporary educational paradigms emphasizing learner autonomy and initiative [8].

Gender Distribution

An equitable distribution of gender among participants was noted, with no significant bias observed in learning styles. This finding suggests that learning styles are not influenced by gender, emphasizing the universality of individual learning preferences irrespective of gender identity. Such gender-neutral findings are essential for promoting inclusivity and equity in educational practices, ensuring that teaching methodologies cater to the diverse needs of all students [9].

Correlation Analysis

Spearman's correlation analysis revealed weak positive correlations between independent, dependent, and participatory learning styles with academic performance, while a weak negative correlation was observed for the avoidant style. These findings suggest that certain learning styles may be modestly associated with academic performance, albeit to varying degrees. The positive correlations observed for independent, dependent, and participatory styles imply that students who exhibit tendencies towards self-directed, collaborative, or active learning may experience incremental gains in academic performance. Conversely, the negative correlation observed for the avoidant style suggests that students who exhibit avoidance behaviours towards learning may experience slight decrements in academic performance. However, it is essential to interpret these correlations with caution, as the strength of the associations is relatively weak, and other factors not accounted for in this study may influence academic performance [10].

No Significant Difference in Academic Performance

Contrary to expectations, the Kruskal-Wallis H Test revealed no significant difference in academic performance scores across different learning styles. This finding suggests that while certain learning styles may be preferred by students, they do not necessarily confer a distinct advantage or disadvantage in terms of academic achievement. This underscores the complexity of the relationship between learning styles and academic performance and emphasizes the need to consider multiple factors that may influence student success, including instructional quality, motivation, and individual aptitude [10].

In comparing similar studies, it becomes evident that there is a consistent interest in understanding the relationship between learning styles and academic success among medical students. For instance, both Smith and Johnson (2023) [11] and Chen and Wang (2022) [12] focused on investigating how different learning styles influence academic performance in medical education. While Smith and Johnson (2023) conducted a detailed exploration of various learning styles and their impact on academic achievement, Chen and Wang (2022) took a systematic review approach to synthesize existing literature on learning style preferences in medical education.

Despite their differing methodologies, both studies converge on the importance of personalized teaching approaches tailored to individual learning styles. Moreover, they highlight the need for a curriculum design that accommodates diverse learning preferences to enhance student engagement and academic outcomes. By comparing and contrasting findings from these similar studies, we gain a more comprehensive understanding of the complex interplay between learning styles and academic success in medical education [13].

Implications for Educational Practice

The findings of this study have significant implications for educational practice within medical education. While learning styles may not exert a direct influence on academic performance, they remain valuable in informing instructional design and pedagogical approaches. Educators should adopt flexible teaching methodologies that accommodate diverse learning preferences, fostering an inclusive and supportive learning environment. [14] Personalized teaching strategies that cater to individual learning styles can enhance student engagement, motivation, and comprehension, ultimately promoting student success in medical education [15].

Limitations and Future Directions

It is essential to acknowledge the limitations of this study, including its cross-sectional design, which precludes causal inference. Additionally, the reliance on self-reported learning styles and cumulative academic scores may introduce response bias and measurement error. Future research should employ longitudinal designs and objective measures of learning styles and academic performance to establish causal relationships and mitigate potential biases. The students exhibit diverse learning styles, yet the assessment relies solely on theoretical and practical examination results.

Moreover, learning styles tend to evolve, and assessing them at a single point in a medical student's life may lack meaningfulness [16]. Furthermore, these scores originate from a single department, whereas learning styles pertain to their overall approach to subjects. Moreover, investigating the role of additional factors, such as instructional quality, study habits, and socio-cultural influences, may provide a more comprehensive understanding of the determinants of academic performance among medical students [17,18].

## Conclusions

While this study provides valuable insights into the relationship between learning styles and academic performance among medical students, several avenues for future research warrant exploration. Longitudinal studies are needed to examine the stability or evolution of learning styles over time and their longitudinal impact on academic outcomes. Additionally, further investigation is warranted to explore the influence of contextual factors, such as curriculum design and teaching methods, on the relationship between learning styles and academic performance. By addressing these gaps in knowledge, future research endeavours can contribute to the ongoing enhancement of teaching and learning practices in medical education.
